# Architecture of Computing System based on Chiplet

**DOI:** 10.3390/mi13020205

**Published:** 2022-01-28

**Authors:** Guangbao Shan, Yanwen Zheng, Chaoyang Xing, Dongdong Chen, Guoliang Li, Yintang Yang

**Affiliations:** 1 School of Microelectronics, Xidian University, Xi’an 710071, China; gbshan@xidian.edu.cn (G.S.); ywzheng@stu.xidian.edu.cn (Y.Z.); ytyang@xidian.edu.cn (Y.Y.); 2 Beijing Institute of Aerospace Control Devices, Beijing 100039, China; 20111110257@stu.xidian.edu.cn

**Keywords:** computing system, computing architecture, memory architecture, Chiplet

## Abstract

Computing systems are widely used in medical diagnosis, climate prediction, autonomous vehicles, etc. As the key part of electronics, the performance of computing systems is crucial in the intellectualization of the equipment. The conflict between performance, efficiency, and cost can be solved by choosing an appropriate computing system architecture. In order to provide useful advice and instructions for the designers to fabricate high-performance computing systems, this paper reviews the Chiplet-based computing system architectures, including computing architecture and memory architecture. Firstly, the computing architecture used for high-performance computing, mobile, and PC is presented and summarized. Secondly, the memory architecture based on mainstream memory and emerging non-volatile memory used for data storing and processing are introduced, and the key parameters of memory are compared and discussed. Finally, this paper is concluded, and the future perspectives of computing system architecture based on Chiplet are presented.

## 1. Introduction

Electronic equipment is becoming more intellectualized with the development of 5G, artificial intelligence (AI), and big data. It has been widely used in medical diagnosis, automotive, electronic product design, Industry 4.0 Internet of things, etc. In the medical field, computer-aided diagnosis can improve efficiency and accuracy by preprocessing and classifying pathological images [1]. Explosive data from vehicles sensors and high-precision avigraph are also processed by computing systems for safety [2]. In addition, computing systems have been used to analyze data from the Internet of Things (IoT) to improve efficiency in smart factories [3]. Precision equipment can be designed by using a computing system; therefore, high-performance computing systems are crucial in electronic equipment [4].

Traditionally, the performance of computing systems can be improved by increasing transistors and frequency of integrated circuits (IC) [5]. In order to meet the requirements of the higher computing power, energy efficiency, and the lower cost of diversified applications, architectural innovation and technology scaling have been proposed to achieve these goals. The computing systems have been developed from single-core to multi-core, including homogenous multi-core and heterogeneous multi-core. In the data-centered applications, the traditional approach is facing some problems: (1) explosive costs [6]; (2) rapid increase in leakage power; (3) scalability degrades; (4) system design complexity increases, which can affect the improvement of the computing system. Due to the advantages of Chiplet, it has been used in the architecture of computing systems [7,8]. Chiplet is a small-scale hard IP with high yield and reusability [9,10,11]. The computing system architecture design based on Chiplet glues together the advantages of technology scaling, three dimensions (3D) integration technology, and a new device to construct a high-performance computing system, which has some merits (1) reducing the design cost via a smaller area and higher yield [12]; (2) avoiding the dark silicon effect [13]; (3) shortening the design cycle by Chiplet reuse; (4) improving the system scalability by flexible Chiplet combinations [14].

**Figure 1:**
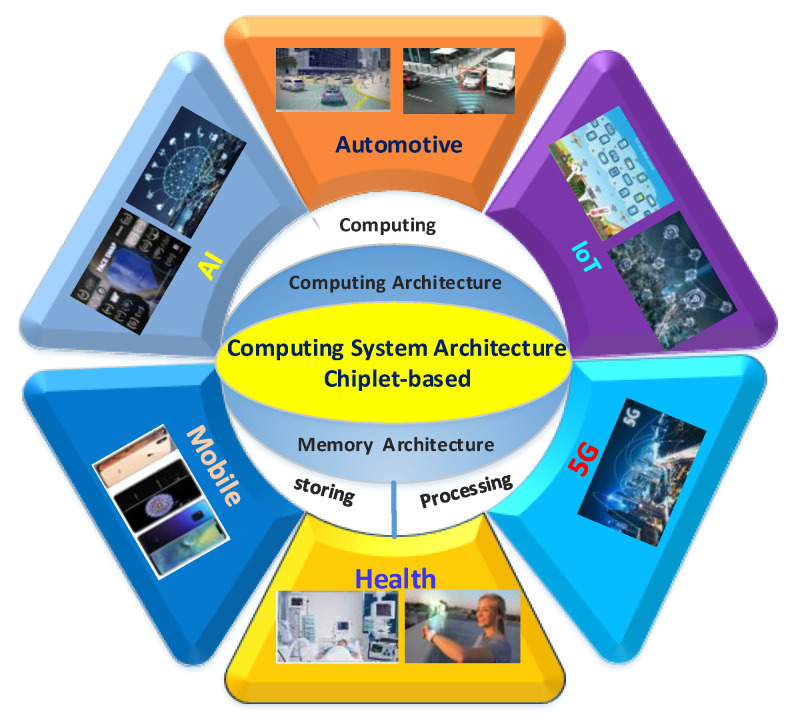
Computing system architectures and application.

The appropriate computing system architecture can effectively utilize the advantages of Chiplet technology in specific applications. This paper aims to summarize the characteristics and performance of Chiplet-based computing and memory architectures to provide instructions for the design of a high-performance computing system. This paper mainly introduces the computing system architectures based on Chiplet, as shown in Figure 1, which mainly includes computing architectures and memory architectures. In computing architecture, 2.5D and 3D computing architectures based on Chiplet are presented and compared. In-memory architecture, near-processor memory architecture, and processing-in-memory architecture based on mainstream and emerging memory are presented and analyzed. Finally, the future perspectives of the computing system architectures based on Chiplet are discussed.

## 2. Computing Architecture Based on Chiplet

**Figure 2:**
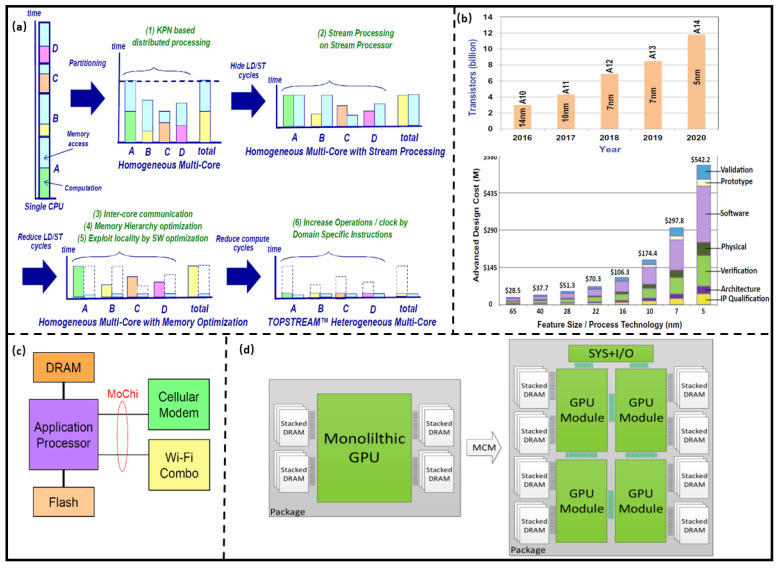
(**a**) Performance comparison of homogeneous and heterogeneous multi-core architectures. (Reprinted from [15], Copyright 2012, with permission from IEEE); (**b**) iPhone processor development and cost analysis. (Reprinted from [16], Copyright 2021, with permission from Springer); (**c**) Mochi Processor architecture. (Reprinted from [17], Copyright 2019, with permission from IEEE); (**d**) GPU design technology based on Chiplet. (Reprinted from [16], Copyright 2021, with permission from Linley Group, Inc.)

As shown in Figure 2a, heterogeneous multi-core architecture has a higher efficiency than single-core and homogenous multi-core architectures [15]. In order to further improve multi-core architectures performance, more transistors were integrated into a limited area of a die; however, the leakage power of the transistor increases as the technology scaling, which severely reduces the energy efficiency of the multi-core architectures. Moreover, in order to ensure the thermal reliability of the computing system, some hardware resources in a die cannot be utilized; that is, the dark silicon effect is more obvious. The architecture of the computing system prepares the computing unit and the memory on one substrate with the same advanced technology, which is a monolithic System on Chip (SoC) and can improve its performance; however, the integration of computing, memory, control, and other IPs into the chip significantly increase the complexity of design and verification. Further, the analog and digital circuits are fabricated using different processes, so multi-manufacturing equipment has to be used in the same process, which dramatically increases the costs. In order to further improve the SoC performance, many chips are designed as dedicated chips, so the chip scalability deteriorates. For example, the performance of Apple mobile SoC processors was significantly improved through technology scaling and architecture updates while the costs obviously raised, as shown in Figure 2b [16].

The computing system architecture designed by modularization and generalization Chiplet can achieve higher performance, lower complexity and cost, and it can reduce the parasitic effect by using 2.5D or 3D integration technology. The computing system architectures based on Chiplet are a key research aspect of computing architecture.

### 2.1. Computing Architecture Integrated with 2.5D Technology

Nurvitadhi et al. [17] compared the performance of GPU (NVIDIA Volta, 10 nm) and Chiplet-based Field Programmable Gate Array (FPGA) integrated with 2.5D integrated technology (Intel Stratix 10, 14 nm) in a given computing task (FP32, INT8). The results show that the computing powers of GPU and Chiplet-based FPGA are 6% and 57% of their peak, respectively. The delay and energy efficiency of FPGA are 1/16 and 34× of GPU, respectively. It shows that the computing system architecture based on Chiplet has higher performance and hardware utilization, as well as lower cost. Sehat [18] proposed the mobile architecture called MoChi, which is integrated by computing Chiplet (such as CPU) with advanced technology and other Chiplets with mature technology. The system resource sharing and communication can be achieved by the MoChi interface, as shown in Figure 2c. The architecture has lower design complexity compared with traditional monolithic SoC architectures. The core of the computing architecture is the integration of Chiplet from different vendors through the interface, and the advanced 2.5D integration technology can be used to reduce the number of pins and packaging costs.

Arunkumar et al. [19] decomposed a single-chip multi-core GPU into multiple GPU Chiplets to design a high-performance computing architecture, as shown in Figure 2d, which can improve computing speed by 22.8% with an energy efficiency of 0.5 pj/bit. The utilization ratio of hardware resources is increased for the GPU and DRAM Chiplet, so the dark silicon effect is alleviated. Further, the yield of the wafer is improved for the larger GPU is decomposed into multiple GPU Chiplet with a smaller area.

**Figure 3:**
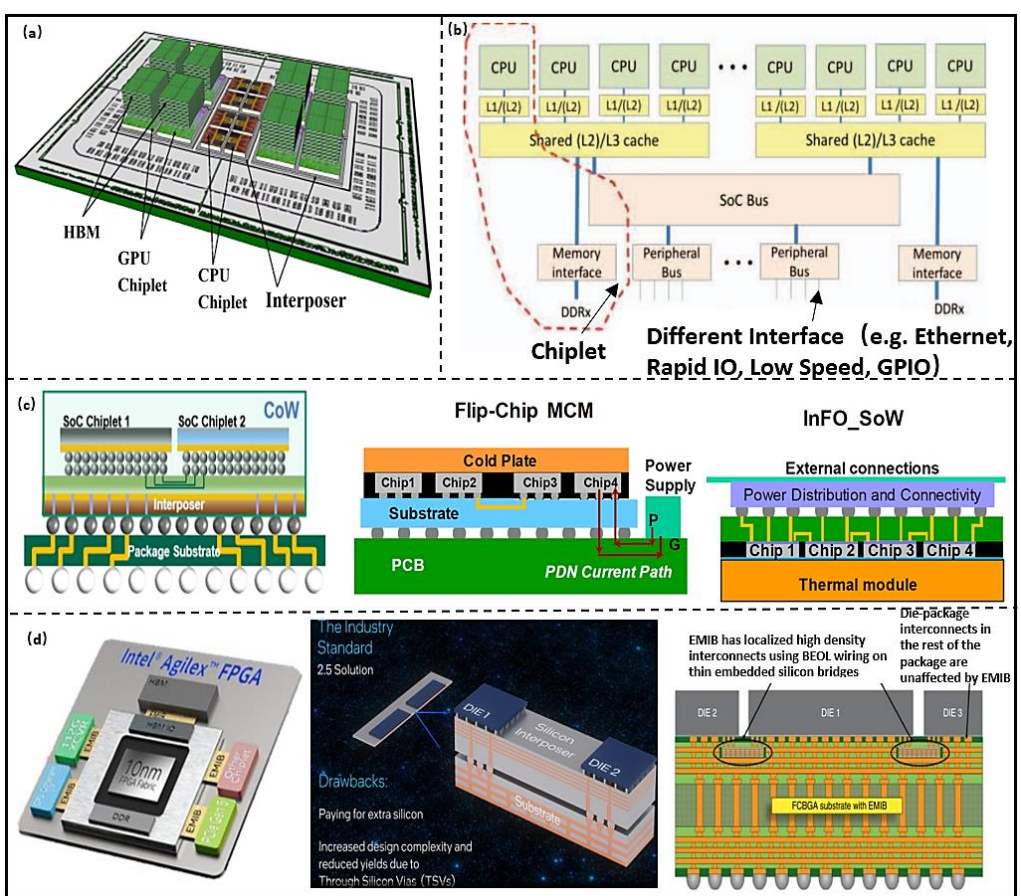
(**a**) Computing system architecture model based on Chiplet. (Reprinted from [19], Copyright 2017, with permission from IEEE); (**b**) Chiplet planning technology. (Reprinted from [20], Copyright 2016, with permission from IEEE) [20]; (**c**) TSMC high performance computing architecture based on Chiplet. (Reprinted from [21], Copyright 2017, with permission from IEEE); (**d**) Chiplet-based integration architecture. (Reprinted from [22], Copyright 2020, with permission from IEEE).

Based on the requirements of the National Aeronautics and Space Administration (NASA) in reconfigurable computing architecture, Mounce et al. [20] proposed a high-performance spatial heterogeneous computing architecture for space applications, as shown in Figure 3b. High-speed communication between Chiplets is implemented by standard communication protocol and bus. In addition, they proposed that the Chiplet-based approach can build more powerful heterogeneous systems with radio frequency (RF) Chiplet and FPGA, and further achieve a smaller size and lower cost. This indicates that the computing system architecture based on Chiplet can take advantage of different hardware resources and achieve higher system scalability. The system performance can be further improved through advanced packaging. Vijayaraghavan et al. [21] designed a Chiplet-based computing system for climate prediction, as shown in Figure 3a. It integrates high-throughput and energy-efficient GPU Chiplet, high-performance multi-core CPU Chiplet, and large capacity 3D memory. The system can achieve a bandwidth of 3 TB/s and power consumption of 160 W at 1 GHz. Lin et al. [22] designed a Chiplet-based high-performance computing architecture, which integrates four 7 nm ARM Cortex-A72 cores in two computing Chiplets. The Chiplet communication can be achieved through the parallel channels formed by Low-voltage-InPackage-INterCONnect technology. The bandwidth rate and density are 320 GB/s and 1.6 Tb/s/mm^2^ under 4 GHz, respectively. The lower roughness and smaller line spacing for the Chiplet connection can be achieved by InFO_SoW technology. The bandwidth density and power distribution network (PDN) impedance are 2× and 30% more than flip-chip multi-chip-module (MCM) interconnection, and interconnection power consumption is reduced by 15% [23], as shown in Figure 3c. In Agilex series FPGAs, the core Chiplet and other Chiplets were interconnected using Embedded Multidie Interconnect Bridge (EMIB). Compared to Stratix10, the delay is reduced by 2.5×, and the bandwidth density and energy efficiency are improved 5.68× and 2.84×, respectively [24]. The interconnect technology has no limitation on the Chiplet area compared to industrial standard 2.5D multi-chip interconnection, which permits flexible placement. The technology can improve the signal and power integrities by isolating signal and power paths, and reducing the cost due to without addition through silicon via (TSV) [25], as shown in Figure 3d. The power consumption of data transfer takes up a large proportion of the total computing system energy. One promising way to improve energy efficiency and bandwidth is to optimize the Chiplet interconnection. The commonality between InFO_SoW and EMIB lies in the preparation of high-density TSV and re-distribution layer (RDL), within the interposer. The Chiplets, interposer, substrate, and printed circuit board (PCB) were integrated by 2.5D technology, so the bandwidth, energy efficiency, signal, and power integrities were improved effectivity.

Zaruba et al. [26] used four computing Chiplets and high bandwidth memory (HBM) Chiplet (8 GB L1 Cache and 27 MB shared L2 memory) to construct computing architecture for high-precision floating-point computing. The computing architecture can be switched between high-performance and high-efficiency modes by reconfiguration. The peak efficiency is larger than 4 TDPflop/s, and power consumption is 25% lower than NVIDIA Volta (7 nm). The efficiency of the architecture is two times and three times that of Intel i9-9900K (14 nm) and ARM N1 (7 nm), respectively. The results show that computing architectures based on Chiplet are more easily integrated with large memory and have a high configurability. Due to the higher modularity of Chiplet, the computing system architecture can be configured in various modes according to the applications. The computing architecture has higher reconfigurability and scalability compared with the traditional SoC-based computing system. It requires co-design of software and hardware, and there is a certain design complexity. Fortunately, there are already solutions for these problems; therefore, the Chiplet-based reconfigurable computing system design technology has obvious technical advantages.

### 2.2. Computing Architecture Integrated with 3D Technology

Since technology scaling cannot improve the performance of digital Chiplet (CPU compute die) and analog Chiplet (IO Chiplet and memory Chiplet) in the same proportion without increasing the cost. The design method of computing architecture based on Chiplet achieves the optimization of performance and cost by selecting the combination of Chiplet with the best technology. Further, it is necessary to reduce the size of electronics driven by small form factors and the lightweight of wearable (motion watch, bodily function devices, etc.), portable electronics (mobile, laptop, etc.); therefore, more and more computing systems are designed with 3D architectures. The computing system performance can be improved by co-design of 3D architectures and advanced packing technology.

**Figure 4:**
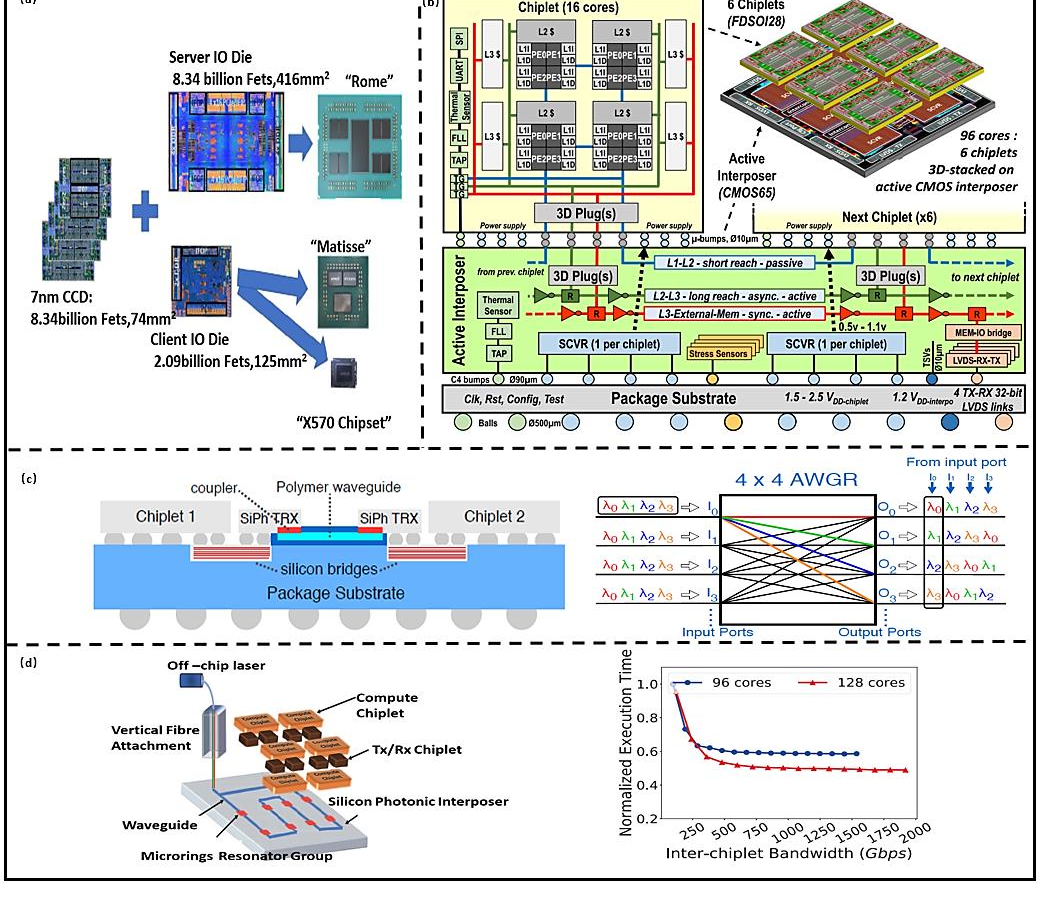
(**a**) AMD processors design technology based on Chiplet. (Reprinted from [9], Copyright 2020, with permission from IEEE); (**b**) INTACT computing architecture based on Chiplet. (Reprinted from [29], Copyright 2019, with permission from IEEE); (**c**) Hybrid optical–electrical interconnection. (Reprinted from [31], Copyright 2020, with permission from IEEE); (**d**) POPSTAR interconnection architecture. (Reprinted from [19], Copyright 2019, with permission from IEEE).

This approach is widely used by AMD in high-performance computing (HPC) system design, enabling rapid development of two products through a different number of Chiplets combinations, such as Rome and Matisse [9], as shown in Figure 4a. The most obvious advantages are that the design of the computing system is simplified and the time to market of product is reduced. The other merits of the architecture include the fact that the digital Chiplet is backward compatible with complex interfaces and the memory Chiplet; that is, the optimal combination of computing and memory Chiplets can be selected according to the computing ability requirements, which has higher scalability and reconfigurability compared with the traditional multi-core architecture and SoC computing system architectures. In order to improve energy efficiency, Kadomoto et al. [27] proposed a method to realize Chiplet communication using the mutual coupling effect of on-chip inductor coils, and fabricated a communication network using 0.18 µm process. The maximum bandwidth can reach 1.6 Gb/s, and the time variation is 3%. The total power consumption is 14.5 mW. The computing architecture has potential in medical microrobots. Although the inter-chip communication based on mutual inductance simplifies the routing design; however, electromagnetic coupling in a small volume leads to signal timing deterioration; therefore, this method requires a sufficient shielding design, which can increase the design difficulty. Burd et al. [28] proposed the infinity fabric (IF) technology to connect Chiplets for higher scalability and configurability in a computing system. It combines scalable data fabric (SDF) and scalable control fabric (SCF) as a critical enabler and utilizes 3D package routing layers to support more complex connections. The in-package bandwidth can achieve 256 GB/s with 534 IFs, and its energy efficiency is 1.2 pj/bit (2 pj/bit for EMIB). CEA-LETI [29] developed a 96-core processor by stacking 28 nm computing Chiplet on the 65 nm interposer with a power management module. The Chiplet interconnected with µbump (20 µm pitch), TSV (depth to width ratio of 10:1 and 40 µm pitch) and RDL (10 µm width and pitch of 20 µm). The Chiplets communication can be achieved by extendable Network on Chip (NoC), and the bandwidth is above 3 Tbit/s/mm^2^, delay below 0.6 ns/mm [30], as shown in Figure 4b. The Lakefield mobile processor also adopted multiple Chiplets design technology, which consists of the computing and memory Chiplets prepared with optimal technology (10 nm and 22 FFL). All Chiplets were bonded face to face with micro-bumps in 50 µm pitch (Foveros technology) [31]. The parasitic capacitance and resistance are below 250 fF and 70 mΩ, respectively. The data transfer rate bandwidth is up to 500 Mb/s with an energy efficiency of 0.2 pj/b. Foveros technology has good compatibility with EMIB and can be used for high-density interconnection of the same system for more flexible interconnection [32]. IF, NoC, and Foveros are all based on 3D electrical interconnection, and the preparation technology is relatively mature. The performance of the computing system is highly predictable. The computing system can obtain a high bandwidth and energy efficiency at a certain working frequency (The typical value is 1.15 GHz, as shown in Table 1); however, with the increase in operating frequency, the parasitic resistor, capacitor, and inductor of TSV and RDL can degrade the signal integrity. In addition, Joule heat produced by TSV and RDL can reduce the system reliability; therefore, more optimized interconnect technologies are needed.

Fotouhi et al. [33] proposed a 3D integration architecture that uses the hybrid Chiplet interconnect technology, as shown in Figure 4c. Silicon bridge is used for a short distance electrical interconnect transceivers (TRXs) Chiplet, and an arrayed waveguide grating router (AWGR) is used for long interconnection in wavelength division multiplexing (WDM). The computing performance is improved by 23%, while the power is reduced by 30%. Narayan et al. [34] designed an optical communication structure for data-parallel transmission between Chiplets by wavelength selection, which can save 38% energy with 1% performance degeneration, and peak bandwidth of 1750 Gb/s, as shown in Figure 4d. AWGR in [34] and interconnection technology in [35] are based on silicon photonic technology, which can realize the selective routing of optical signals by adjusting wavelengths. The higher data bandwidth, smaller signal delay, less heat, and higher energy efficiency can be achieved compared with the electrical interconnection; however, silicon photonic communication requires a high-power laser source, which is difficult to be integrated on the chip. In addition, the performance of optical devices is greatly affected by the fluctuation of the process, so the reliability is lower than the electrical interconnection. Due to the difficulty of fabrication and integration of silicon photonic devices, optical interconnection technology cannot be widely used; however, the advantages of the technology will drive the development of the integration technology, and it will be more widely used in future computing systems.

### 2.3. Summary

Single-core and homogeneous multi-core architectures handle task parallelization and computing acceleration under lightweight workloads. Heterogeneous computing architectures can improve energy efficiency by integrating the merits of different computing cores, such as CPU–GPU/CPU–NPU; however, multi-core architectures cannot improve computing performance and energy efficiency as further increasing intensive workloads and scaling of technology and the dark silicon effect are made worse as cores increase in number. It can achieve a single optimization for performance, energy efficiency, or scalability. In the Chiplet-based computing system, the Chiplet is prepared with the optimized technology and further integrated with 2.5/3D advanced packing technology, which has high bandwidth and energy efficiency and low data delay. As shown in Table 1, in [22], the computing architecture was constructed with the four Chiplets using 2.5D Chip on Wafer on a substrate (CoWoS) technology, and the bandwidth can be improved to 1.6 Tb/s/mm^2^ in high-performance computing. In [24], the delay of Agilex can be reduced to 60 ps by using 2.5D integration technology, and the architecture has high configurability and reusability. In [25], the energy efficiency of Lakefield can be improved to 0.2 pJ/b, and the architecture can be configured for PC and mobile processors. In [28], the Chiplets were prepared with the most mature technology among all computing systems; however, the delay can be reduced to 0.6 ns/mm and the bandwidth can be improved to 527 GB/s through 3D integration. In [26], the interconnect pitch between μbumps can be reduced to 20 µm through 2.5D integration, and the maximum bandwidth reaches 1 TB/s. Due to the mature preparation technology of electrical interconnection and higher energy efficiency of silicon photon interconnection, these two technologies have obvious application advantages in Chiplet-based computing system architecture.

The Chiplet-based 2.5D and 3D integrated architectures have obvious advantages; however, the diversified applications have different focuses. In terms of data bandwidth, the 3D integrated architecture is better, which requires better thermal design. This architecture is more suitable for high-performance computing, for example, data center, networking, server, etc. In terms of cost, the 2.5D integrated architecture does not require a multi-layer Interposer with high-density TSVs; thus, the process is less difficult. The architecture is more suitable for applications such as mobile, laptop, wearable electronics, etc. In terms of Chiplet materials, due to the same thermal expansion coefficient, multiple homogeneous Chiplets adopt the 3D integrated architecture, which is beneficial to improve mechanical reliability; heterogeneous Chiplets are more suitable for the 2.5D integrated architecture (such as EMIB integration technology), which has the higher performance of system heat dissipation, while its area will be increased.

## 3. Memory Architecture Based on Chiplet

**Figure 5:**
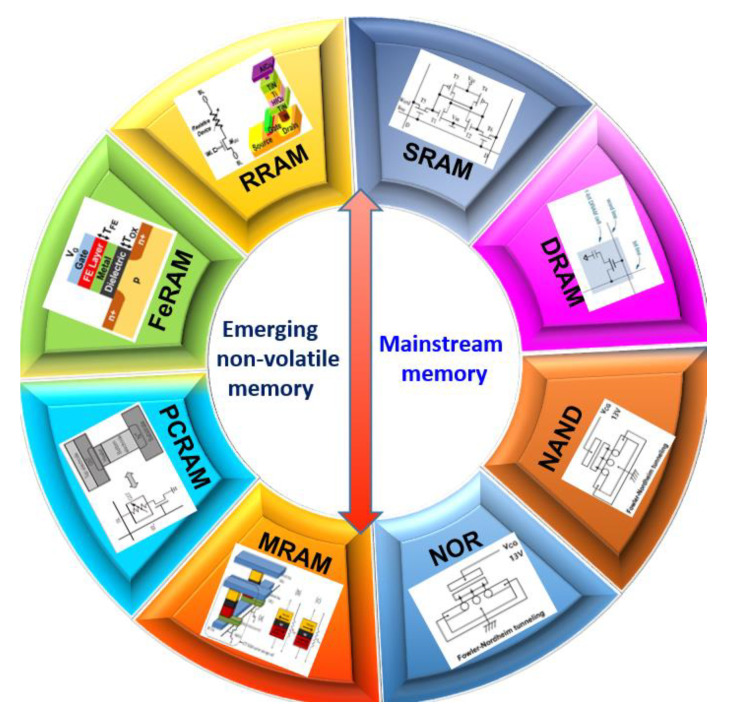
Memory architecture based on different memory. Note: SRAM (Static Random-Access Memory); DRAM (Dynamic RAM); MRAM (Magnetoresistive Random Access Memory); PCRAM (Phase Change RAM); FeRAM (Ferroelectric RAM); RRAM (Resistive RAM).

The explosive data eagerly demands memory with larger capacity, bandwidth, and energy efficiency [35,36,37]; however, the mainstream memory has the relatively matured preparation technology, while the poor integration density and energy efficiency, the emerging memory is just the opposite. Thus, the problems can be solved by optimizing the current memory architecture and introducing emerging non-volatile memory. This section introduces mainstream memory architecture and emerging non-volatile memory architecture, as shown in Figure 5.

### 3.1. Memory Architecture for Storing Data

**Figure 6:**
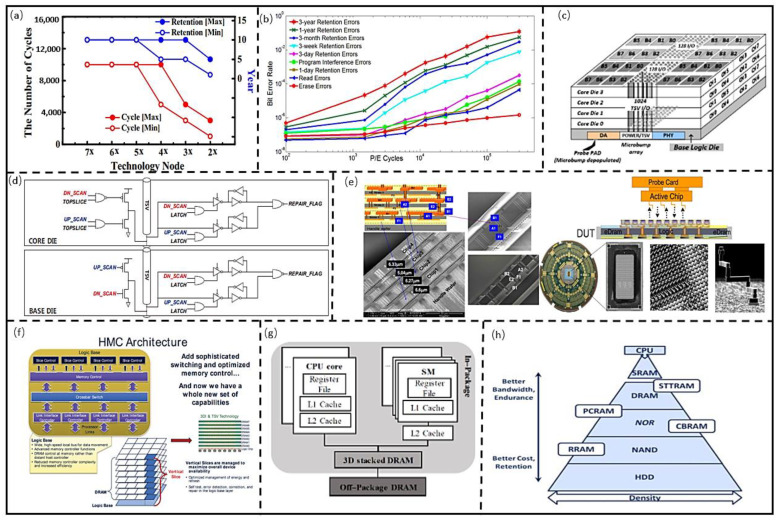
(**a**) Flash performance change with technology. (Reprinted from [40], Copyright 2019, with permission from IEEE); (**b**) NAND performance vary with process technology. (Reprinted from [41], Copyright 2012, with permission from IEEE); (**c**) HBM memory architecture. (Reprinted from [43], Copyright 2017, with permission from IEEE); (**d**) HBM interconnect architecture optimization [44]; (**e**) 3D DRAM Architecture and test architecture (Reprinted from [45], Copyright 2016, with permission from IEEE); (**f**) micron hybrid memory architecture. (Reprinted from [46], Copyright 2011, with permission from IEEE); (**g**) 3D computing systems integrates memory and sensor. (Reprinted from [47], Copyright 2017, with permission from Nature); (**h**) computing systems with hybrid memories (Reprinted from [48], Copyright 2013, with permission from IEEE).

Due to the big cell area, 2D architectures for mainstream storage have a large form factor, which cannot meet the minimization of electronics. Moreover, the cost of mainstream memory is increasing as the technology shrinks while its endurance is decreasing [38,39]. Koh et al. [40] proposed Flash retention, which is decreased as the technology scales, as shown in Figure 6a. Cai et al. [41] found the error proportions in NAND rising with the increase in write/read cycles, as shown in Figure 6b.

Therefore, the 2D architecture of mainstream storage cannot meet the needs of high-performance storage, and the mainstream storage architecture needs to be optimized. Loi et al. [42] proved 3D memory architecture has a smaller delay than 2D architecture with a bus model, and the performance is significantly improved in intensive applications, as the frequency increases. Jun et al. [43] designed the 3D HBM architecture for data storage in parallel computing, as shown in Figure 6c. The 2N-Prefetch data mechanism was also used for improving bandwidth (up to 256 GB/s) in data acquisition. Lee et al. [44] further optimized the 3D memory data channel, which can increase the bandwidth of HBM 1 and HBM 2 by 4.6× and 9.1× compared with DDR5, and its power is reduced by 42%. They also proposed the self-repair structure of TSV for increasing testability and reliability, as shown in Figure 6d. Thus, the mainstream memory with 3D architecture can effectively improve bandwidth; however, another difficulty in 3D architecture is the test structure of the memory. Kirihata et al. [45] designed 3D DRAM using TSV for high-density interconnection and developed the electromechanical system (MEMS) probe card for rapid detections, as shown in Figure 6e. A wider and faster bus for data movement was proposed by Micron to simplify the memory control mechanism, which can avoid the complex scheduler and deep queue [46], as shown in Figure 6f. The energy efficiency and bandwidth are 10.82 pj/bit and 128 GB/s, respectively. Shulaker et al. [47] designed a computing system for integrated storage, calculation, and perception of the Chiplet. The 3D integration architecture was adopted to reduce the transmission distance between the data in the Chiplet and improve the signal and power integrities. The whole system was developed by CMOS technology with low preparation difficulty, as shown in Figure 6g. Sandhu et al. [48] proposed a hierarchical memory system, as shown in Figure 6h; it combines the merits of non-volatile memory (NVM) and mainstream memory to improve bandwidth and decrease power.

### 3.2. Memory Architecture for Processing Data

The energy for data transfer between memory and computing is about 4× that of computing in Von Neumann computing architecture, which reduces the energy efficiency significantly [49,50]. Processing-in-memory (PIM) can complete the data computing and storage in memory with high power efficiency in computation-intensive applications [51,52,53]. In addition, 3D memory architecture shortens the data transmission path by vertically stacking multiple Chiplets compared with 2D storage architecture and effectively reduces the energy consumption and improves the thermal reliability [47].

#### 3.2.1. PIM Architectures Based on Mainstream Memory

**Figure 7:**
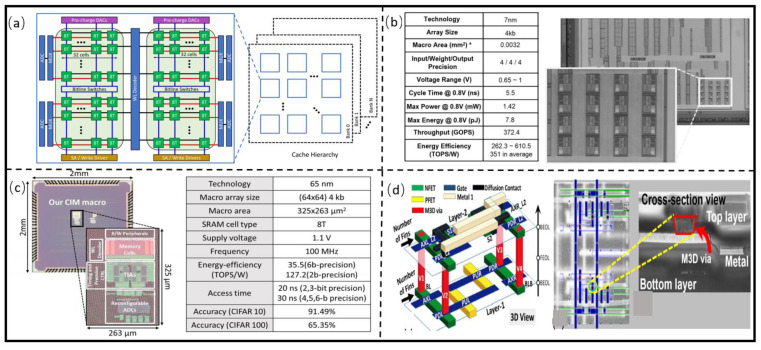
(**a**) An 8T SRAM PIM Chiplet. (Reprinted from [55], Copyright 2020, with permission from IEEE) (**b**) 3D SRAM PIM Chiplet. (Reprinted from [56], Copyright 2021, with permission from IEEE); (**c**) reconfigurable SRAM PIM Chiplet. (Reprinted from [57], Copyright 2021, with permission from IEEE); (**d**) 3D SRAM PIM Chiplet architecture and SEM photo. (Reprinted from [58], Copyright 2019, with permission from IEEE).

Agrawal et al. [54] designed an 8 TB SRAM Chiplet, which uses parasitic capacitance for accumulating voltages and dot product calculation. The energy-delay product (EDP) is 38% lower than that of Von Neumann computing systems within the acceptable accuracy degradation range (1–5%), as shown in Figure 7a. Sinangil et al. [55] developed the SRAM PIM architecture, which can simultaneously perform multiply and sum computation with the average energy efficiency of 3511 TOPS/W, as shown in Figure 7b. They prepared the SRAM Chiplet with an area of 0.0032 mm^2^ using 7 nm technology. Ali et al. [56] designed and prepared a 65 nm SRAM PIM Chiplet, which dynamically uses sparsity of workload to configure the output precision of peripheral circuits to keep data accuracy, as shown in Figure 7c. The energy efficiency is above 120 TOPS/W at 1.1 V, 100 MHz. Srinivasa et al. [57] designed SRAM PIM Chiplet with 3D architecture, as shown in Figure 7d. The read and write stabilities are improved 6.6% and 17.6%. The read and write delay times are reduced 17.5% and 6.6%, and EDP is decreased by 1.6× compared with baseline. The design and preparation technology of SRAM Chiplet is relatively mature. As shown in Table 2, it has the fastest read and write speed and the lowest read and write power consumption; however, the cell is large since it requires four or six transistors to store 1 bit of data. Moreover, the volatility of SRAM requires a continuous power supply, and the transistor generates high static power consumption, which hinders its widespread application.

**Figure 8:**
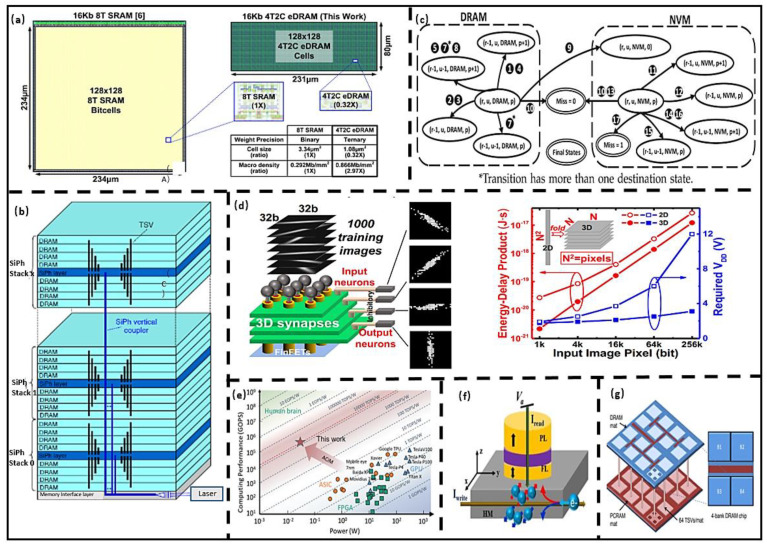
(**a**) Embedded DRAM Chiplet. (Reprinted from [58], Copyright 2021, with permission from IEEE); (**b**) 3D DRAM based on optical interconnect. (Reprinted from [59], Copyright 2019, with permission from IEEE); (**c**) Chiplet evaluation technology. (Reprinted from [61], Copyright 2019, with permission from IEEE); (**d**) 3D RRAM Chiplet, (Reprinted from [62], Copyright 2020, with permission from IEEE); (**e**) FeRAM Chiplet vs. traditional computing system. (Reprinted from [65], Copyright 2020, with permission from IEEE); (**f**) MRAM Chiplet (Reprinted from [68], Copy-right 2021, with permission from ELSEVIER); (**g**) 3D PCRAM Chiplet, (Reprinted from [68], Copy-right 2009, with permission from IEEE).

Yu et al. [58] designed the embedded DRAM PIM Chiplet for vector-matrix operation in a neural network, as shown in Figure 8a. In the proposed architecture, the memory node capacitance is increased to improve retention time, which can improve the system energy efficiency up to 552.5 TOPS/W. Werner et al. [59] used vertical optical interconnects (VOIs) to connect the DRAM Chiplet, which eliminates heavily coupling between TSVs, as shown in Figure 8b. Ali et al. [60] designed a DRAM Chiplet to perform data operation in odd rows simultaneously. It improves the parallelism of operation and data throughput, and its performance improves 11.5× compared with baseline. Salkhordeh et al. [61] proposed an analysis model based on the Markov decision method to evaluate the hit ratio and the average lifetime of hybrid memory (DRAM-NVM), as shown in Figure 8c. Compared to the latest simulator, the error is decreased by 2.93%, and speed is improved by 10×. It is a promising way to use the parasitic capacitor of DRAM Chiplet to improve retention time as well as signal quality. Since DRAM needs to be constantly refreshed, an efficient evaluation method is needed to predict the reliability of DRAM Chiplet, and Markov evaluation technology has merits in evaluation efficiency.

#### 3.2.2. PIM Architectures Based on Emerging Nonvolatile Memory

The RRAM architecture designed by Liang et al. [62] could be reconstructed into logical and memory modes. They further developed the adaptive layout and routing algorithms to improve efficient utilization. The power consumption and delays of the proposed architecture are reduced by 1.9× and 2.8×, respectively, and its performance is improved by 5.6× compared with FPGA. Li et al. [63] designed the 3D PIM architecture based on RRAM Chiplet, as shown in Figure 8d, which uses four Chiplets for stacked, and ferroelectric field effect transistor (FeFET) is used as selectors. The voltage, EDP, and area are reduced by 74%, 55%, and 4× compared to 2D memory, respectively. RRAM has good compatibility with CMOS technology and is suitable for high-density integration; however, as a logic Chiplet, the conductive filaments in its structure are affected by the randomness of metal atoms, which generates random noise in the logic mode. The RRAM Chiplet is more suitable as memory. Due to the unique characteristics of hysteretic, FinFET can be designed either as a switch or memory. Yin et al. [64] designed a PIM Chiplet based on FeRAM, whose area and power consumption are 58% and 64% of the SRAM, respectively. Soliman et al. [65] prepared an FeRAM Chiplet with 28 nm CMOS technology; the energy efficiency and latency are 13714 TOPS/W and 0.5 ns, respectively, when 2-bit data operations are performed, as shown in Figure 8e. FeRAM has low read/write time and power consumption and has the best compatibility with CMOS technology; however, the FeRAM Chiplet has a high cost because its electrode materials are noble metals (Pt, Ir). Angizi et al. [66] designed the Chiplet-based MRAM to solve the multi-period logic problem in PIM architecture. Its energy efficiency and speed are 1.7× and 11.2× than those of ASIC, respectively. Shreya et al. [67] designed the Spin-Orbit Torque MRAM PIM Chiplet based on the voltage control technique. The power and data transfer energy consumption are reduced 53.98% and 2.7%, respectively, compared with traditional structures. The read and write time and current of MRAM are small, and it is expected to be used as an L2 cache, that is, to supplement the existing cache. Dong et al. [68] proposed a 3D PCRAM Chiplet used for checkpointing in parallel computing, which incurs less than 6% overhead in an exascale computing system by making near-instantaneous checkpoints, as shown in Figure 8g. The PCRAM Chiplet requires a large write current to melt the phase change material. As shown in Table 2, the data retention is affected by the amorphous resistance drift of the phase change material, and the power consumption and speed are inferior to RRAM.

### 3.3. Summary

Due to its mature design and manufacturing technology, the mainstream memory Chiplet has been widely used in IoT, PC, mobile, etc. With the technology scaling, the current memory architecture design is dealing with issues regarding a compromise of bandwidth, capacity, power consumption, and cost. As shown in Table 2, due to the shortest read/write time (1 ns), the SRAM Chiplet is used as a cache; however, the cell area is above 160 F^2^, which hinders the miniaturization of memory systems. Because of the mature design and fabrication techniques, the SRAM Chiplet is still used as a small capacity, fast read/write storage (cache). The working voltage and cell area of MRAM and DRAM are similar (voltage: 1 V, 1.5 V, cell area: 10 F^2^). The static current of MRAM is smaller than that of DRAM, and it can be used as the main memory. NAND and NOR Flash are preferred for large-capacity memory due to their long read/write and lower cost. The PCRAM and RRAM are expected to complement the existing large capacity storage with a smaller static current (~10^−4^ A), and their cell areas are similar to that of NOR and NADN (10 F^2^, 4 F^2^). The low read/write energy of FeRAM makes it more promising in low-power applications; however, the current immature technology seriously affects the volume manufacture of NVM. Thus, the Chiplet-based 3D integration technology is an effective method to design high-performance memory. The 3D PIM architecture based on mainstream memory and emerging memory can effectively reduce the distance of data movement, and complete data storage and calculation at the same time, which has obvious application advantages in data-centric computing systems.

## 4. Conclusions and Perspectives

In this paper, the Chiplet-based computing system architectures with 2.5D and 3D integration technology are introduced, and their characteristics and performance indexes are summarized. The mainstream and emerging NVM memory architectures are also introduced, and their structures and key parameters are summarized. The advantages and disadvantages of the three computing architectures, including single-core, multi-core, and Chiplet-based, are summarized and compared, and their applications are shown. The single-core computing system architecture has a short design cycle and low cost and is mainly applied to light-load computing. Multi-core computing system architecture can meet the requirements of multi-task parallelization and high-precision computing; however, the performance improvement gradually slows down as technology scaling, and the dark silicon effect is obvious. The Chiplet-based computing system architecture has merits of high scalability, energy efficiency, and low cost. With the driven by diversified applications, these computing system architectures will develop in parallel, and the Chiplet-based architectures design method will gradually become the mainstream method of computing system architecture in HPC Mobile, etc. The future development and perspectives for the computing system based on Chiplet are summarized as follows:

(1) Advanced integration technology. The Chiplet-based 2.5D and 3D integration technologies will be widely used in high-performance computing systems. The AI-based optimization layout technology for Chiplet can not only improve the integration density but also enhance the thermal routing capability of computing systems.
(2) Standardized interconnection protocols. The standardized interconnection protocols can achieve the normalization and modularization of Chiplet in computing systems, which can decrease the research and development cycle and cost for Chiplet-based computing systems.
(3) Scalable and reconfigurable architecture design technology. The scalable and reconfigurable technology can effectively improve the utilization efficiency of Chiplet, and then improve the utilization range of computing systems, which can also decrease the research and development cycle and cost.

## Figures and Tables

**Table 1:** Comparison of computing architectures based on Chiplet.

	Intel [24]	TSMC [22]	AMD [9]	CEA-Leti [30]	Intel [25]	Bologna [26]
Product Name	Agilex	-	Ryzen	INTACT	Lakefield	Manticore
Launched Time	201904	201908	201908	202002	202006	202012
Chiplet Technology (nm)	10	7	7 + 12	FDSOI 28	10 + 22 FFL	GF 22 FDX
Chiplet Number	scalable	2	>2	6	1	4
Number of cores/Chiplet	Cortex-A53	4 Cortex-A72	64 (Server) 16 (Cilient)	16	1 Core+ 4 Atom	1024 RISC-V
Area (mm^2^)	-	4.4 × 6.2	-	4 × 5.6	-	9
Bandwidth (Max)	32 Gb/s	320 GB/s	~55 GB/s	527 GB/s	~34 GB/s	1 TB/s
Bandwidth density		1.6 Tb/s/mm^2^	-	3 Tbit/s/mm^2^	-	-
Frequency (GHz)	1.5	4	~1	1.15	~1	1
Integrated type	2.5D	2.5D	3D	3D	3D	2.5D
Interposer type	Passive	Passive	N/A	Active	Active	Yes
Interconnect pitch (µm)	55	40	-	20	50	20
Delay	~60 ps	-	<9 ns	0.6 ns/mm	-	-
Integration technology	EMIB	CoWoS		F2F	Foveros	-
Yield	High	High	High	High	High	High
Scalability	High		High	High		-
Configurability	Good	Yes	Yes	Yes	alternative	High efficiency/performance
Reusability	High	High	High	High	High	High
Testability			Good	Good	Good	
Power efficiency	-	0.56 pJ/b	2 pJ/b	0.59 pj/b	0.2 pJ/b	50 Gdopflop/sW
Application	Data Center, Networking, Edge Computing	HPC	Server and Desktop Products	Cloud Computing Accelerators	Mobile, PC	Data Center, Networking, Edge Computing.

**Table 2:** Memory Chiplet comparison.

	SRAM Chiplet [69,70]	DRAM Chiplet [71,72]	NOR Chiplet [69]	NAND Chiplet [73]	MRAM Chiplet [74]	PCRAM Chiplet [75,76]	RRAM Chiplet [77,78]	FeRAM Chiplet [67]
Technology [79]	7 nm	14 nm	28 nm	32 nm	28 nm	28 nm	28 nm	-
Cell area	160–280 F^2^	10 F^2^	10 F^2^	4 F^2^	10–20 F^2^	5–20 F^2^	4–10 F^2^	15–20 F^2^
Voltage (V)	<1	~1	~10	~15	<1.5	<2	1–3	~1
Current (A)	~10^−5^	~10^−5^	~10^−7^	~10^−7^	~10^−5^	~10^−4^	~10^−4^	~10^−6^
Read time (ns)	~1	~10	~10	~10	~10	~10	~10	<10
Write time	~1 ns	~10 ns	10 μs–1 ms	~1 ms	~10 ns	~50 ns	~10 ns	~10 ns
Write energy	~fj	~10 fj	~100 pj	~10 pj	0.1 pj	~10 pj	~0.1 pj	~0.1 pj
Endurance	~10^16^	~10^16^	~10^5^	~10^5^	~10^15^	~10^9^	10^6^–10^12^	~10^10^
Retention	N/A	~64 ms	>10 y	>10 y	>10 y	>10 y	>10 y	>10 y
Static power	High	High	Medium	Medium	Low	Low	Low	Low
Dynamic power	Low	Low	Medium	Medium	Medium	Medium	Medium	Medium
Anti-radiation	Low	Low	Very low	Very low	High	High	High	High
No volatility	NO	NO	Yes	Yes	Yes	Yes	Yes	Yes

Note: F is feature sizes.
